# When Positron Emission Tomography (PET) Is Misleading: Ion™ Robotic Bronchoscopy Identifies Organizing Pneumonia and Unmasks a Rare Pulmonary Granular Cell Tumor

**DOI:** 10.7759/cureus.103084

**Published:** 2026-02-06

**Authors:** Tashfeen Mahmood, Robert L Rumsey, Mohammad M Mahmood, Rameesha Y Awan

**Affiliations:** 1 Pulmonology, Critical Care and Sleep Medicine, Christus Ochsner St. Patrick Hospital, Lake Charles, USA; 2 Pathology, The Pathology Laboratory, Lake Charles, USA; 3 Science, Bishop Noland Episcopal Day School, Lake Charles, USA; 4 Independent Physician, Hull Royal Infirmary, York, GBR

**Keywords:** cryptogenic organizing pneumonia (cop), false positive pet scan, ion endoluminal system, peripheral pulmonary nodule, pulmonary granular cell tumor

## Abstract

Pulmonary nodules with increased metabolic activity on positron emission tomography (PET) are frequently presumed malignant; however, inflammatory and rare benign neoplastic processes may produce false-positive findings. A 61-year-old African American male with a history of calcified and non-calcified pulmonary nodules and severe emphysema was referred to our pulmonary nodule clinic from the emergency department after a newly discovered lung nodule was identified on computed tomography (CT) of the chest. Subsequent evaluation demonstrated metabolic activity on PET, raising concern for malignancy; however, tissue diagnosis revealed organizing pneumonia. This case highlights diagnostic pitfalls associated with commonly used imaging and biomarker modalities in the evaluation of pulmonary nodules and emphasizes the importance of clinical awareness of a rare tumor, granular cell tumor (GCT), among physicians who may be unfamiliar with or have never encountered this condition.

## Introduction

Granular cell tumor (GCT) is an exceptionally rare neoplasm, accounting for approximately 0.5% of all soft-tissue tumors, with only about 1-2% demonstrating malignant behavior [1]. GCTs are uncommon tumors derived from Schwann cells and most frequently occur in the skin, tongue, breast, and gastrointestinal tract [1,2]. They are neoplasms of neuroectodermal origin and are histologically characterized by large polygonal cells with abundant granular cytoplasm [1,3,4]. Although pulmonary involvement is rare, GCTs may be encountered incidentally during the evaluation of lung lesions [3]. In contrast, organizing pneumonia is a benign inflammatory process that may present as a focal pulmonary lesion with increased metabolic activity on positron emission tomography, thereby mimicking malignancy [5,6]. This report addresses a common diagnostic dilemma in pulmonary nodule evaluation, i.e. false-positive PET avidity and discordant adjunct testing and highlights the clinical implications of confirming diagnosis by tissue sampling while maintaining awareness of rare benign endobronchial tumors.

## Case presentation

**Figure 1:**
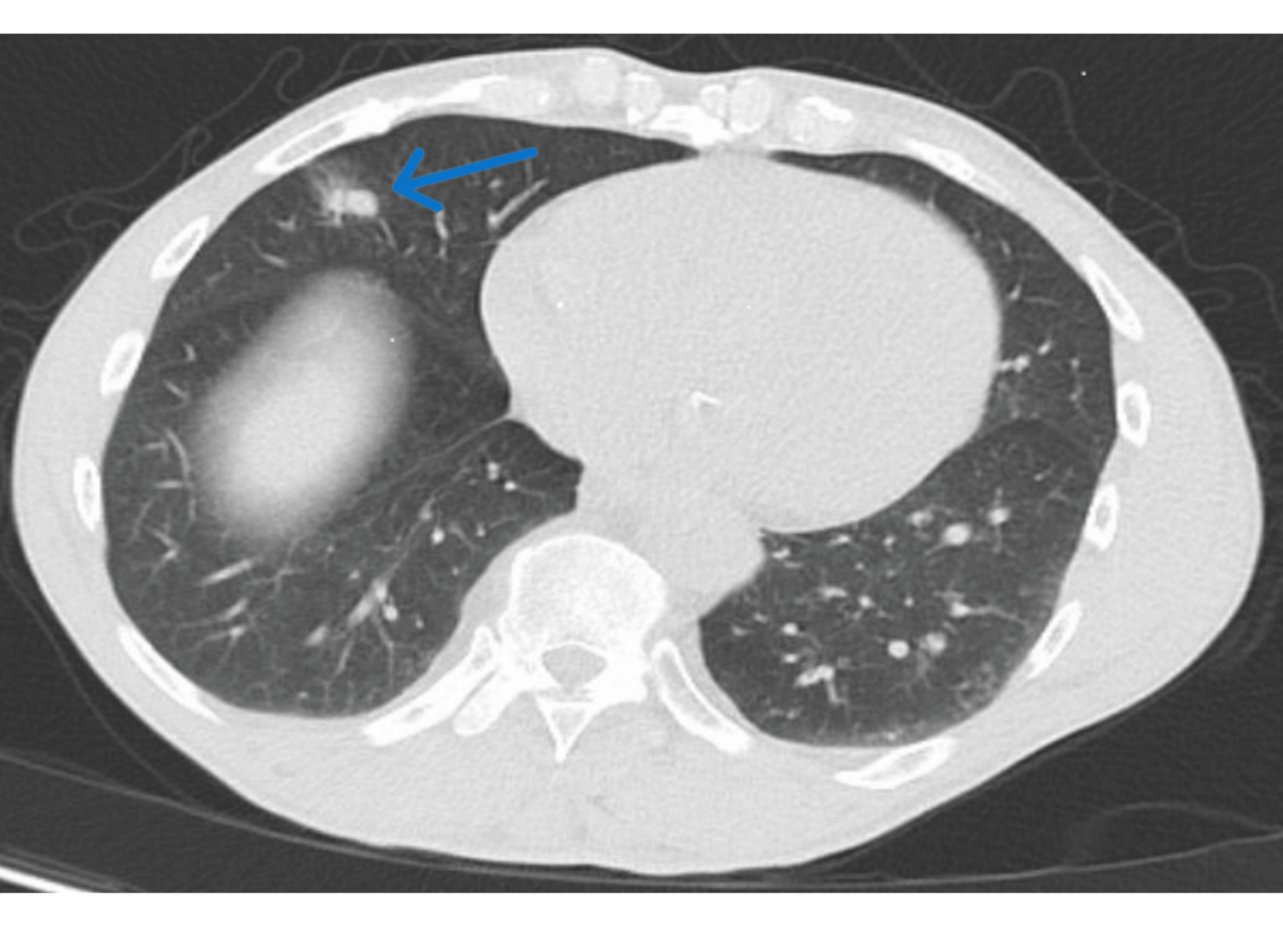
CT angiogram of chest demonstrating right middle lobe part solid pulmonary nodule (blue arrow).

A 61-year-old African American man presented to the emergency department with chest pain after a friend opened a car door, causing a load of cargo to fall onto his chest. A CT angiogram of the chest was obtained due to elevated D-dimer levels and demonstrated severe emphysema with a newly identified part-solid right middle lobe pulmonary nodule (Figure 1). A summary of key findings at presentation and initial workup is provided in Table 1 (with corresponding figure references).

**Table 1:** Summary of key findings at presentation and initial workup (with corresponding figure references). ED: Emergency department; FDG: fluorodeoxyglucose, RML: right middle lobe, CDT: Nodify® Combined Diagnostic Test (CDT), rEBUS: radial endobronchial ultrasound, LLL: left lower lobe.

Domain	Key findings	Figure/Notes
Presentation	Chest pain after blunt chest trauma; no systemic symptoms reported.	ED evaluation
CT angiogram	Severe emphysema; new 1.5 cm part-solid right middle lobe nodule.	Figure 1
Prior nodules	Stable tubular/slightly lobulated nodules consistent with mucus impaction.	Figure 2
PET/CT	Moderate FDG uptake in RML nodule (SUVmax 4.2); no other avid lesions.	Figure 3
Biomarkers	CyPath low-risk (0.11%); Nodify-CDT moderate risk (25%).	Text
Bronchoscopy	ION robotic bronchoscopy with rEBUS and intraoperative 3D CT; incidental LLL endobronchial lesion.	Figure 4
Pathology	LLL: granular cell tumor without malignant features.	Figures 5–9
Historical pathology	Remote (1993) cutaneous neck lesion biopsy consistent with granular cell tumor.	Records review

**Figure 2:**
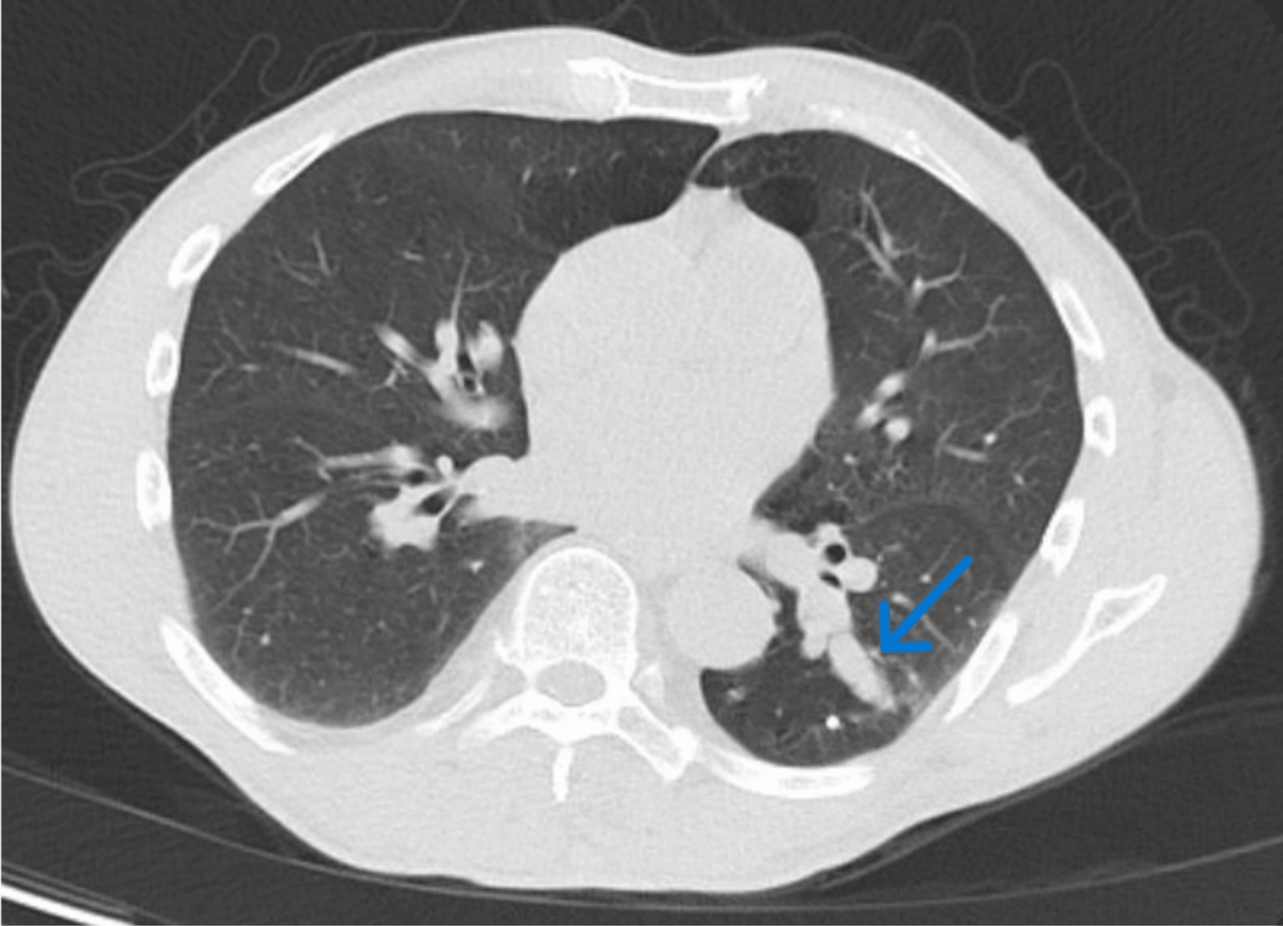
CT chest demonstrating scar-like, mucus-impaction appearance (blue arrow) of left lower lobe lesion.

The patient was referred to our pulmonary clinic for further evaluation. He had previously been followed by another pulmonologist with serial CT imaging for several stable nodules described as tubular-shaped and slightly lobulated, thought to represent mucus plugging (Figure 2). He denied fever, chills, night sweats, weight loss, loss of appetite, or hemoptysis.

His past medical history included anemia, gastroesophageal reflux disease, hyperlipidemia, hypertension, and colonic polyps. Past surgical history was notable for a Whipple procedure, colonoscopy, and foot reconstruction.

**Figure 3:**
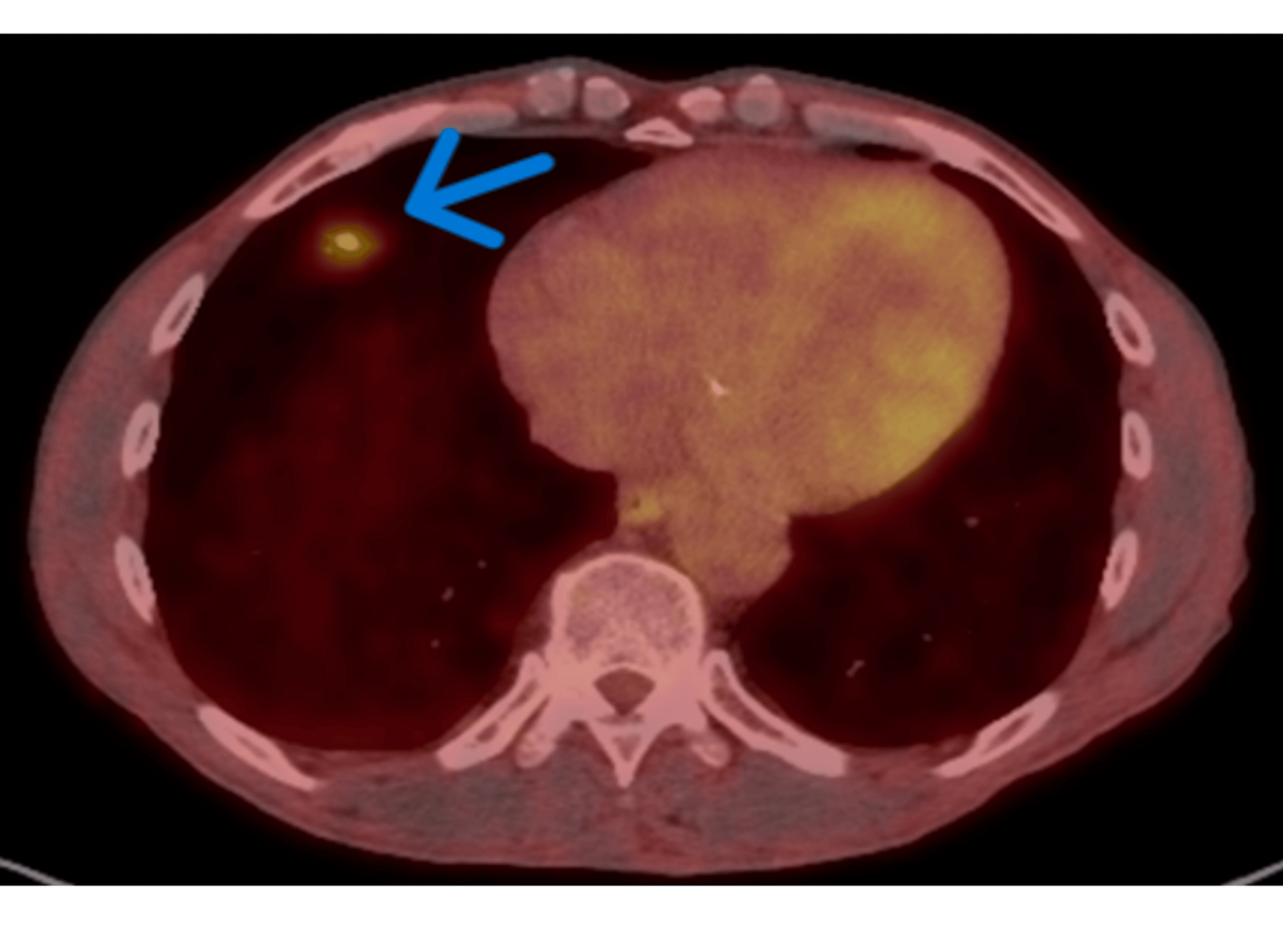
Fluorodeoxyglucose (FDG) PET/CT showing moderate uptake in right middle lobe nodule (blue arrow) (SUV max 4.2). SUV: Standardized uptake value.

Further evaluation included positron emission tomography (PET) imaging, Nodify®-CDT±XL2 (Biodesix, Louisville, CO, USA), and CyPath® testing (bioAffinity Technologies, San Antonio, TX, USA). CyPath® demonstrated a 0.11% risk of malignancy, while Nodify®-CDT showed a moderate (25%) risk for malignancy, warranting further evaluation. PET imaging revealed a hypermetabolic 1.5-cm part-solid nodule in the right middle lobe, raising concern for malignancy (Figure 3). No other nodules demonstrated increased metabolic activity.

The discordance between advanced biomarker testing and PET imaging raised concern for a false-positive PET result and prompted tissue diagnosis. Fluorodeoxyglucose (FDG) uptake on PET imaging reflects increased glucose metabolism and is not specific for malignancy, as inflammatory processes such as organizing pneumonia may demonstrate increased metabolic activity and mimic malignancy [5,6].

**Figure 4:**
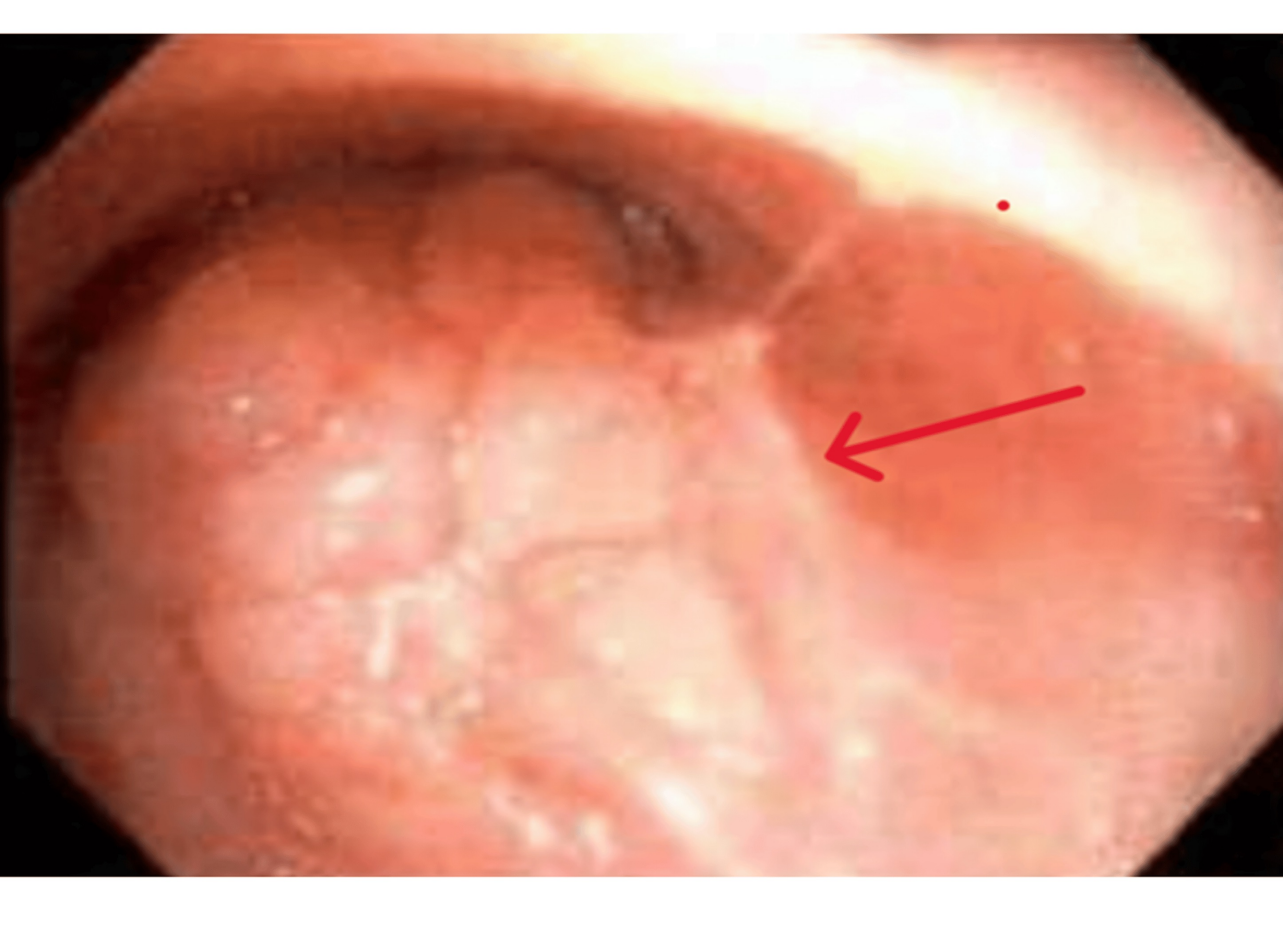
Bronchoscopic view demonstrating a smooth, submucosal endobronchial lesion in the left lower lobe (red arrow), later confirmed on cryobiopsy to represent a granular cell tumor without malignant features.

The patient underwent robotic-assisted bronchoscopy using the Ion™ Robotic Bronchoscopy System (Intuitive Surgical, Sunnyvale, CA, USA), augmented with intraoperative three-dimensional CT imaging and radial endobronchial ultrasound. Transbronchial needle aspiration and cryobiopsy of the right middle lobe lesion were performed. A detailed bronchoscopic survey revealed a smooth, submucosal endobronchial lesion in the left lower lobe (Figure 4). This lesion was biopsied using cryoprobe.

**Figure 5:**
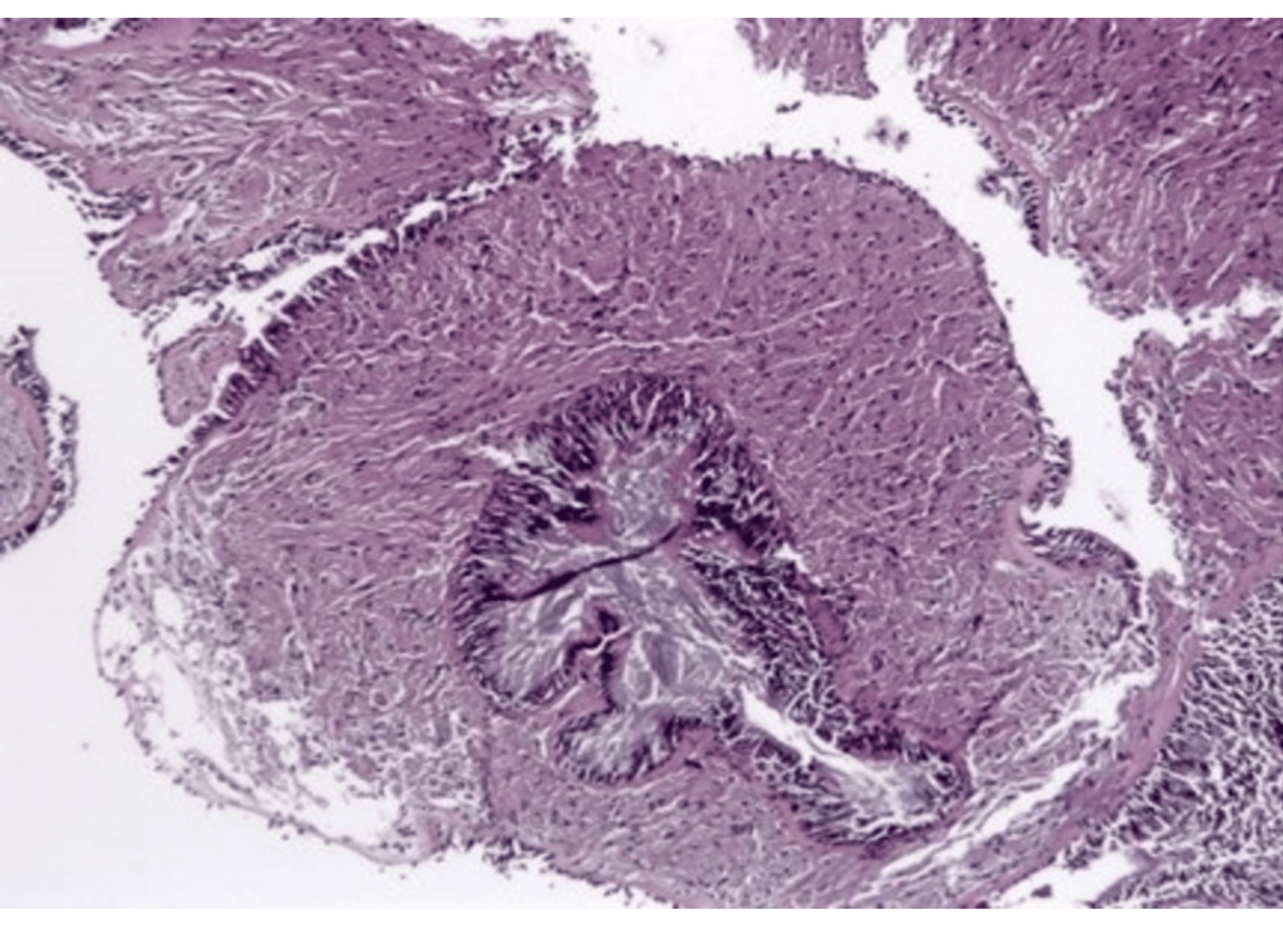
Low-power hematoxylin and eosin (H&E) showing submucosal, poorly circumscribed lesion surrounding bronchial wall with preserved epithelium.

**Figure 6:**
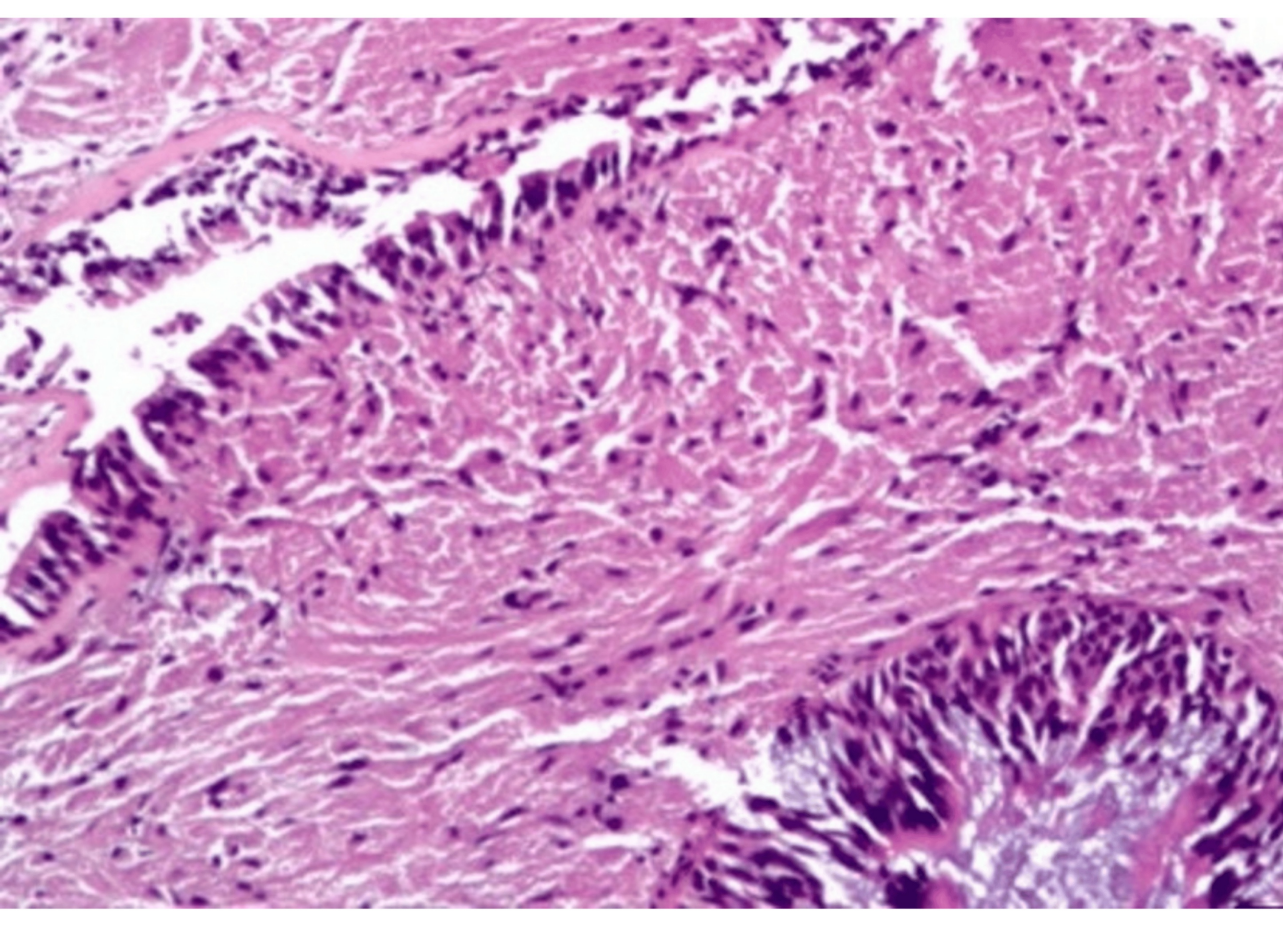
High-power hematoxylin and eosin (H&E) demonstrating polygonal cells with abundant eosinophilic granular cytoplasm and small, uniform nuclei without atypia.

**Figure 7:**
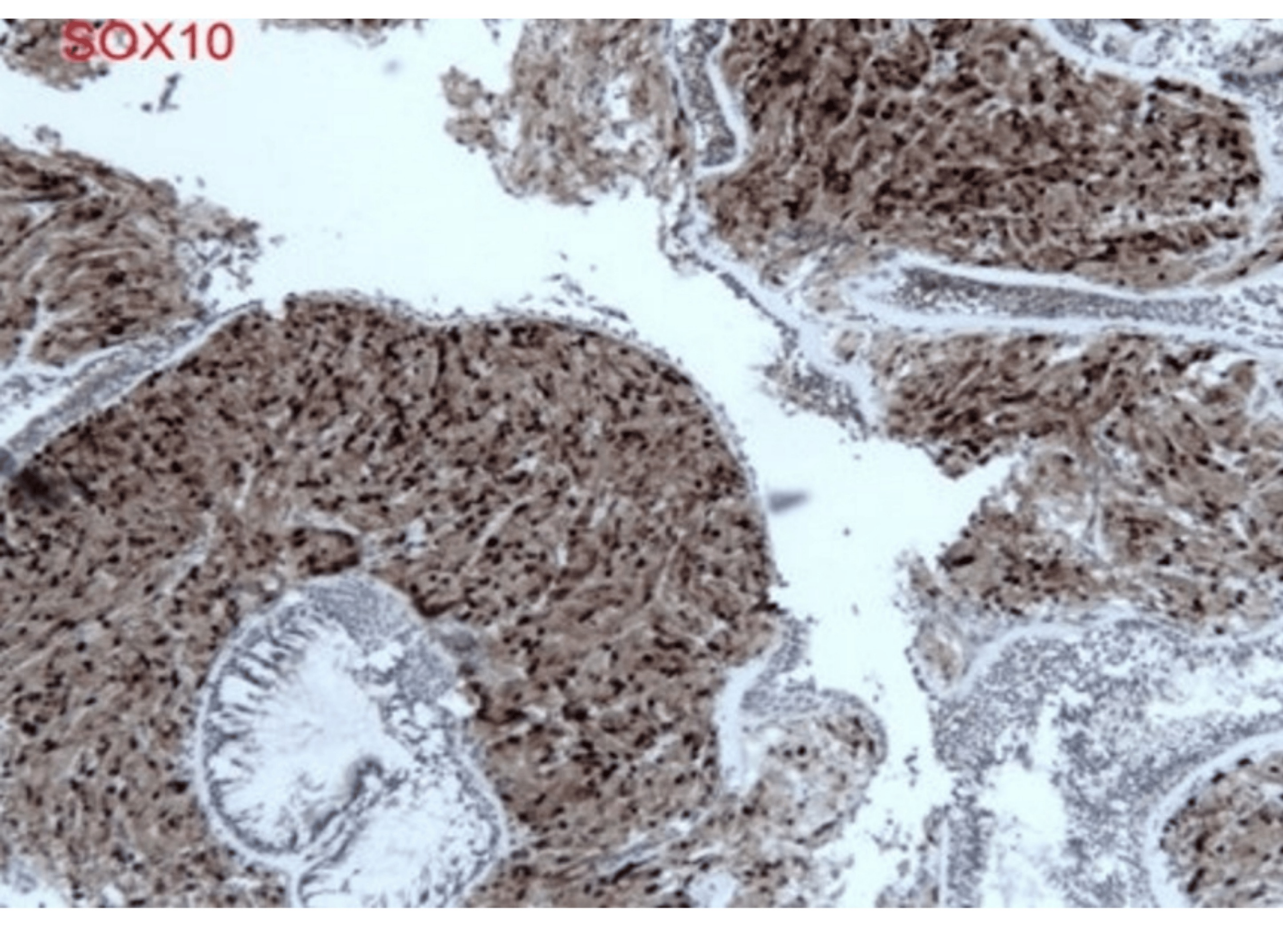
Diffuse immunoreactivity for SOX10

**Figure 8:**
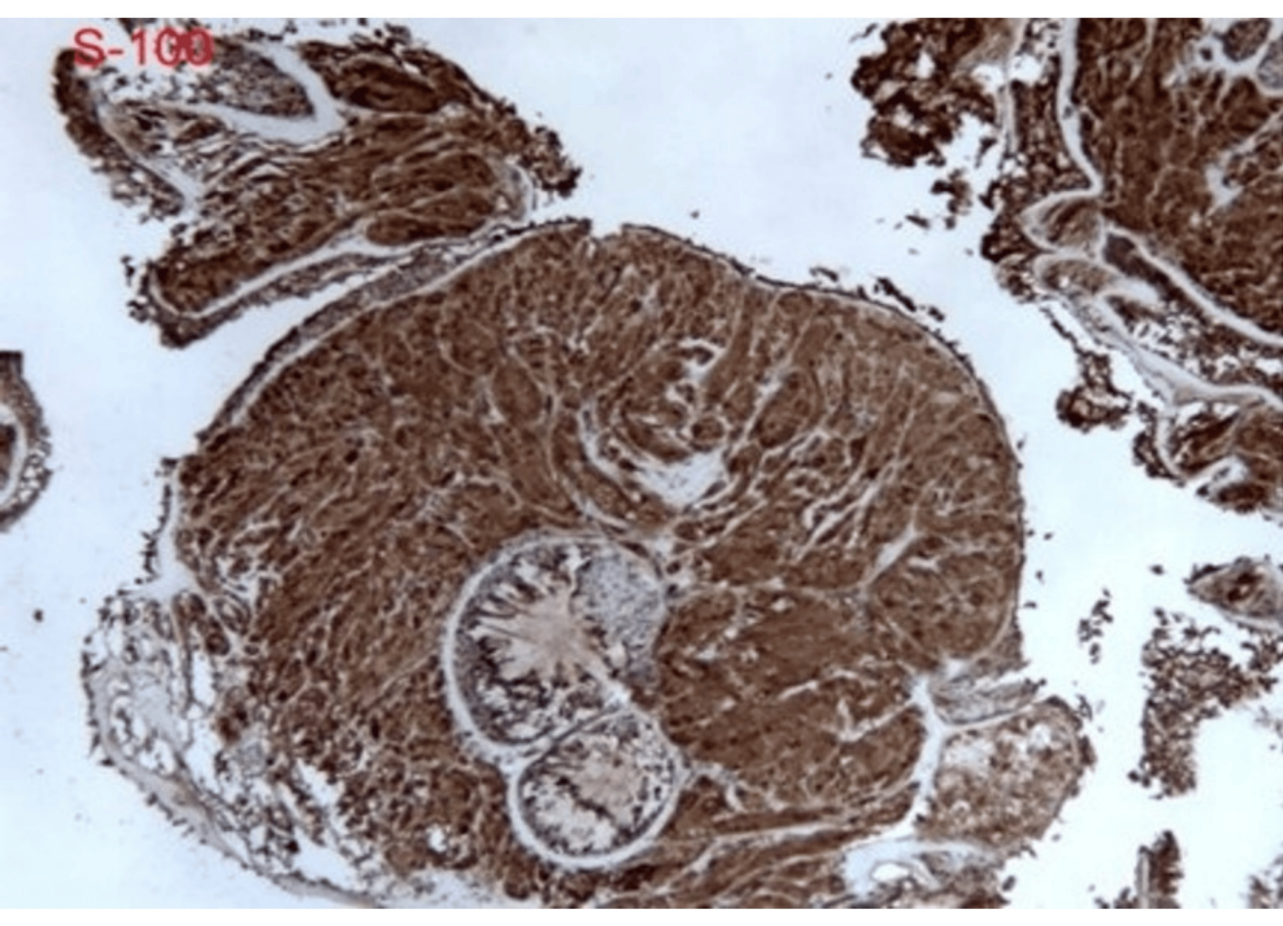
Diffuse immunoreactivity for S-100

**Figure 9:**
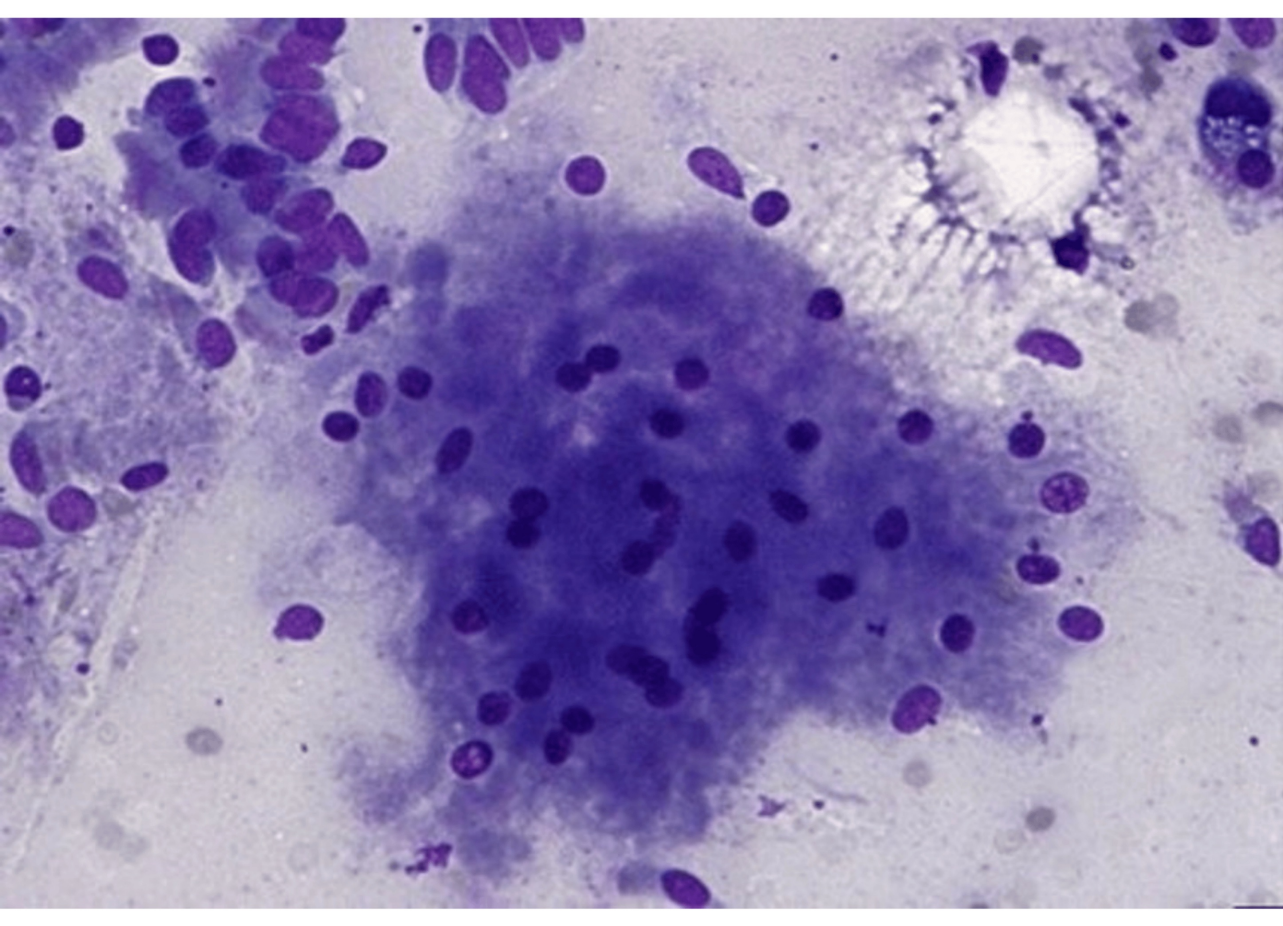
Cytology specimen with loosely cohesive clusters of cells showing granular cytoplasm and bland nuclei.

Pathologic evaluation of the right middle lobe lesion was consistent with organizing pneumonia. In contrast, biopsy of the left lower lobe endobronchial lesion demonstrated findings consistent with granular cell tumor without malignant features, including polygonal cells with abundant granular cytoplasm and supportive immunohistochemical staining (Figures 5-9) [1,3].

Upon further review of prior medical records, the pathologist identified a remote biopsy performed in 1993 for a cutaneous lesion on the patient’s neck, which had also been histologically consistent with a granular cell tumor. The patient did not recall this prior diagnosis at the time of presentation. This finding suggests long-standing multifocal disease rather than a de novo pulmonary process [2].

## Discussion

Granular cell tumors are rare neoplasms that most commonly occur in the skin, oral cavity, and gastrointestinal tract, with less frequent involvement of the respiratory tract, nervous system, and breast [1]. These tumors are believed to originate from Schwann cells and have been associated with certain genetic syndromes, including Noonan syndrome and LEOPARD syndrome (LEOPARD=Lentigines, Electrocardiographic conduction abnormalities, Ocular hypertelorism, Pulmonary stenosis, Abnormal genitalia, Retardation of growth, and Deafness) [1,2].

Most granular cell tumors are diagnosed in middle-aged individuals, with a higher prevalence reported among patients of African descent [1,2]. A significant proportion are discovered incidentally, particularly in the gastrointestinal tract and skin during evaluation for unrelated conditions [1]. This incidental nature, combined with their typically indolent growth pattern, may contribute to under-recognition or delayed diagnosis.

Granular cell tumors may occur as multifocal lesions, either synchronously or metachronously, and patients may not recall prior diagnoses given the slow-growing and often asymptomatic course of the disease [2]. In the present case, retrospective review of historical pathology revealed a granular cell tumor diagnosed from a cutaneous neck lesion in 1993, supporting long-standing multifocal disease rather than a de novo pulmonary process. This finding underscores the importance of a thorough pathologic correlation and detailed historical record review when evaluating newly discovered lesions suspicious for malignancy.

Although granular cell tumors are typically slow-growing and painless, clinical manifestations vary depending on the tumor location. Endobronchial involvement may lead to cough, hoarseness, recurrent pneumonias due to obstructive physiology, or dysphagia, potentially mimicking more common malignant or inflammatory airway processes [1,3]. This case further illustrates how reliance on imaging and biomarkers alone may lead to diagnostic anchoring, underscoring the continued importance of tissue confirmation. Awareness of this rare entity is therefore essential to avoid misdiagnosis and unnecessary interventions.

## Conclusions

This case raises awareness of a rare pulmonary granular cell tumor and underscores the importance of tissue diagnosis in the evaluation of pulmonary nodules. It also highlights the limitations of PET imaging and adjunctive biomarker testing, such as Nodify®-CDT and CyPath®, which may yield misleading results in the presence of inflammatory conditions. The identification of a remote cutaneous granular cell tumor further emphasizes the potential for multifocal disease and the importance of comprehensive clinical and pathologic correlation.
